# Prevalence of overweight and its associated factors among selected higher secondary schools adolescents of Kathmandu Metropolitan City, Nepal

**DOI:** 10.1371/journal.pone.0334832

**Published:** 2025-10-17

**Authors:** Karishma Bhandari, Sheetal Bhandari, Manish Rajbanshi, Richa Aryal, Sagun Magar, Lokendra Oli, Mohandev Joshi, Bishnu Prasad Choulagai

**Affiliations:** 1 Central Department of Public Health, Institute of Medicine, Tribhuvan University, Kathmandu, Nepal; 2 School of Public Health and Community Medicine, B.P Koirala Institute of Health Sciences, Dharan, Nepal; 3 Department of Psychiatry, Padma Kanya Multiple Campus, Tribhuvan University, Kathmandu, Nepal; 4 Ministry of Health and Population, Government of Nepal, Kathmandu, Nepal; Siddhi Memorial Hospital, NEPAL

## Abstract

Overweight is highly prevalent in lower- and middle-income countries (LMICs), including Nepal. Due to the rapid physical and mental growth among adolescents, they are nutritionally vulnerable and sensitive to environmental factors and dietary habits. This study aimed to determine the prevalence of overweight and its associated factors among adolescents of higher secondary schools in Kathmandu Metropolitan City (KMC) of Nepal. A cross-sectional study was conducted among 282 adolescents in higher secondary schools. A stratified random sampling technique was used to select the participants for data collection. The frequencies, percentages, mean, and standard deviation were used to describe the characteristics of the participants. Binary logistic regression was performed to determine the association between individual characteristics and the prevalence of overweight. All the tests were performed at a 95% Confidence Interval (CI), and variables with p-values below 0.05 were considered statistically significant. The mean (±SD) age of the participants was 16.8 ± 0.1 years. The majority of the participants (66.3%) were from private schools. Around 13.4% of the participants were overweight. Characteristics such as type of school (AOR: 2.6, CI: 1.9–8.2), father’s education (AOR: 2.1, CI: 1.7–6.5), access to physical activity at school (AOR: 1.2, CI: 1.1–4.6), and pocket money for lunch at school (AOR: 0.3, CI: 0.2–0.5) were found to be significantly associated with overweight among adolescents in this study. This study found that a notable proportion of adolescents were overweight and were influenced by socio-economic and demographical characteristics such as education, income level, school type, and level of physical activity. School-based interventions and programs should be carried out to promote healthy eating and physical activity among adolescents. A holistic approach, including parental education on nutrition, controlling pocket money to reduce unhealthy purchases, and adding physical activities to school programs, should be tailored to the school setting to reduce the risk of being overweight.

## Introduction

Overweight is defined as abnormal or excessive fat accumulation that can harm one’s health [1]. The etiology of adolescent overweight is multifaceted, involving a complex interplay of genetic, behavioral, socio-cultural, and environmental factors [2]. The World Health Organization (WHO) defines “adolescents as those people between 10-19 years of age” [3]. Adolescents are not only a transition period to adulthood but also one of the most dynamic stages of human development, where they start to make individual choices and develop behaviors that often persist in adulthood [4,5]. Due to their high development requirements, dietary habits, and sensitivity to environmental factors, adolescents are nutritionally vulnerable [6]. Being overweight during adolescence can lead to immediate health problems and increase the risk of developing non-communicable diseases (NCDs) like type 2 diabetes, hypertension, and heart disease at earlier stages [7,8]. It also has negative effects on mental and emotional well-being, impacting school performance and overall quality of life due to stigma, discrimination, and bullying [1].

Despite being among the most evident government health issues, being overweight is often ignored [9,10]. In 2022, one in eight people in the world was overweight, and adolescent overweight has quadrupled since 1990 [1]. Meanwhile, over 390 million children and adolescents aged 15–19 years were overweight, and 160 million were obese in 2022 [1]. It was predicted that one in ten adolescents would be affected by these risk factors by 2030 [11,12]. It was estimated that the majority of children and adolescents (57.3%) will suffer from overweight by age 35 by 2050 [13]. All these findings warrant extraordinary responses from all countries, particularly developing countries, to combat the risk of overweight among adolescents.

Overweight is highly prevalent in LMICs, including Nepal [14]. This is due to rapid globalization and urbanization, which have led to a reduction in energy expenditure along with an increased intake of high-fat, energy-dense food and a sedentary lifestyle [15]. A systematic review and meta-analysis conducted in Asian countries found that the prevalence of overweight among adolescents (12–19 years) was 14.6% [12]. Various studies have reported the prevalence of overweight among adolescents as 18.3% in Ajman, Sharjah, and Dubai [16], 19% in Delhi [17], and 13.5% in Bangladesh [18].

In Nepal, the fourth stage of the nutrition transition has begun, resulting in a rapid increase in overweight [19]. According to the Nepal Demographic Health Survey (NDHS) 2022, 2.1% of the adolescents (15–19) years were overweight/obese [20]. Nepal National Micronutrient Survey Status (NNMSS) 2016 showed that 5% and 4% of adolescent boys and girls were obese [21]. The Adolescent Nutrition Survey in Nepal 2014 reported that 94% of adolescents consumed fast or junk food, with 92% consuming it at least once a week [6]. A study conducted in the urban district of Nepal, Rupandehi, found that about 31% of adolescents did not meet the WHO recommendation of 60 minutes of moderate to vigorous physical activity per day [22]. To address the rising issue, Nepal has committed to one of the key targets of the Sustainable Development Goal (SDG) 3.4 to reduce premature mortality from NCDs by one-third through prevention and treatment by 2030 [23].

A major modifiable risk factor for the rise in overweight among adolescents is physical inactivity, along with poor eating habits [24,25]. Once the adolescent becomes obese, it becomes difficult to reduce the excessive weight [26,27]. Thus, early identification of risk behaviors and implementation of preventive measures are crucial for preventing NCDs and future health complications among adolescents. The study results would be helpful for policymakers, governments, development partners, and other stakeholders to design effective health programs tailored to adolescents’ needs and the prevention of risk factors against overweight. Therefore, this study aimed to assess the prevalence of overweight and its associated factors among adolescents of selected higher secondary schools in KMC of Nepal. This study examined how socio-demographic factors, school and home physical environments, physical activity, dietary habits, and behavioral characteristics are associated with the prevalence of overweight among adolescents

## Materials and methods

### Study design and setting

A cross-sectional study was conducted in KMC, located in the Kathmandu District of Bagmati Province. This metropolitan city is divided into 32 wards and has a literacy rate of 90.5% as per the 2021 Census. A total of 470 higher secondary schools (HSS) is located within the city, comprising both government and private schools.

There was a total population of 862400, among them, more than 16% were contributed by adolescents (Census, 2021) [24]. This study was carried out in 4 private and 2 government higher secondary schools [28]. The study of overweight and obesity among adolescents in urban settings like Kathmandu District is essential due to the growing burden of behavior-related risk factors influenced by rapid urbanization and changing lifestyles. Exposure to unhealthy dietary patterns, physical inactivity, prolonged screen time, and limited access to recreational spaces underscores the need for evidence-based interventions to mitigate future NCDs risks in this vulnerable population.

### Study population

The study included adolescents in grades 11 and 12 from selected higher secondary schools of KMC. The students belonging to the age group between 14–19 years and enrolled in selected government and private higher secondary schools were included in the study. Participants with disabilities related to hearing, mental, and speech problems were not included in this study. Also, absentee students at the time of data collection were excluded from the study.

### Sample size and sampling technique

The sample size was calculated using the Cochrane single proportion formula, i.e., n = (Z^2^pq/d^2^) [29]. The proportion of adolescents (p) being overweight (8.8%) was taken from a previous study conducted by Shakya et al, 2023 in Nepal. Assuming a 5% margin of error (d), 95% Confidence Interval (CI), 10% non-response rate, and using a design effect of 2, the minimum obtained sample size was n = 273.

**Fig 1:**
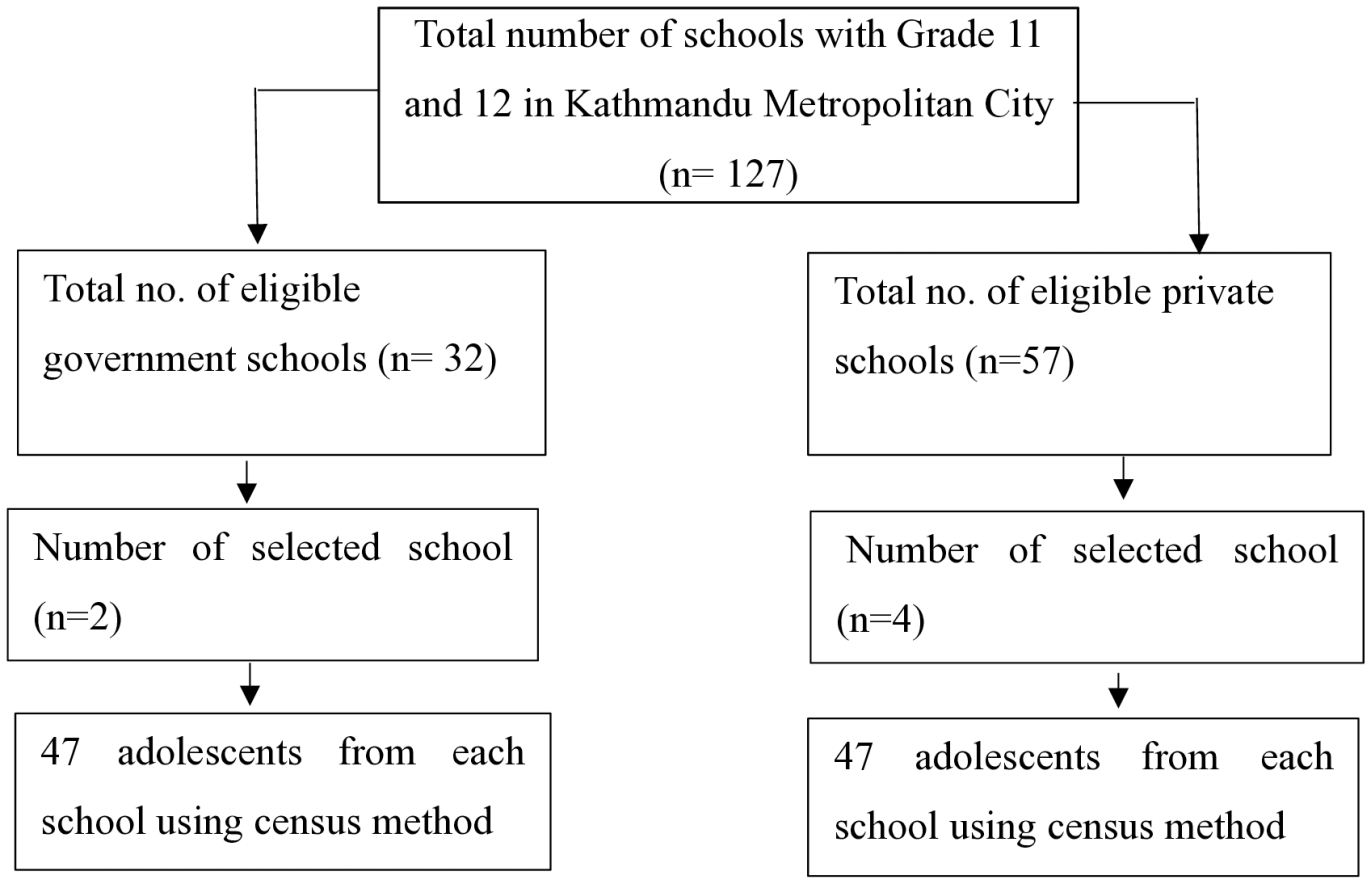
Sampling framework.

Detailed information on total HSS was obtained from the Education Department of KMC. Altogether, there were 32 government and 57 private HSS in KMC. Then, 6 schools (2 government and 4 private) were selected using a stratified random sampling technique. The government and private schools were considered as strata. The government and private schools consisted of 34% and 66% of the total students, respectively. The required number of students for each selected government (n_1_ = 94) and private school (n_2_ = 183) was calculated based on population size, i.e., proportionate sampling technique. A systematic random sampling technique (N/n)^th^ item was used to select participants from the selected classroom. The first participant was selected randomly by lottery method, and every 3^rd^ participant was taken in the study (Fig 1).

### Tools and measures

The study tool was adapted from the Nepal WHO STEP-wise approach to the NCD risk factor surveillance (STEPS) Survey in 2019 [30]. The validated tool in the Nepali language was used in the study. Further, pretesting was conducted to check internal consistency, feasibility, and necessary modifications. The pretesting was done among 10% of the sample size (n = 28) of the Tokha Municipality of Kathmandu District, which was not included in the study. After obtaining a Cronbach’s Alpha coefficient of 0.85, a tool was used for the data collection.

The tool was divided into four sections. It included;

**Section I:** This section included questions regarding social, economic, and demographical characteristics of the participants, such as sex, age, religion, ethnicity, birth order, school type, family type, mothers’ and fathers’ education, mothers’ and fathers’ occupation, and house type.
**Section II:** It consisted of questions related to physical and environmental factors such as the availability of playgrounds in school, provision of extracurricular activities at school, availability of playgrounds at home, and means of transportation to go to school.
**Section III:** It consisted of questions related to food habits such as main diet, frequency of meal consumption, meal skipping, frequency of consumption (green leafy vegetables, fruits, meat, fish, and egg), frequency of fast-food consumption, frequency of junk food consumption, frequency of soft/sweetened drinks consumption.
**Section IV:** It consisted of questions related to physical activity and anthropometric measurement. The physical activity consisted frequency of vigorous-intensity activities, the frequency of moderate-intensity activities, sedentary behaviours, and screen time. Anthropometric measurement consisted of the measurement of the height and weight of the participants to determine their Body Mass Index (BMI). Participants’ height was measured with a stadiometer, and weight with a digital scale (SECA) as recommended by UNICEF [31].

### Data collection and techniques

A data collection was conducted between 26^th^ January to 30^th^ March 2024. A self-administered questionnaire was used for data collection. The Principal Investigator (KB) and Research Assistants were responsible for data collection. Research assistants were provided with orientation regarding the study project, data collection procedure, and handling of the instruments for data collection.

Participants’ height was measured by using the following procedure: participants were asked to remove footwear and caps, and requested to stand on a flat surface with their feet together and knees straight [30]. Weight was measured by using the following procedure: The weighing machine was placed firmly on a flat surface. Participants were asked to remove footwear with minimal possible during the measurement [30]. Both height and weight were measured twice, and the average values were computed in this study.

### Data management and analysis

Collected data was systematically entered, compiled, coded, filtered, and cross-checked in EpiData software version 3.1 and exported to IBM Statistical Package for Social Sciences (SPSS) version 26 for data analysis. The descriptive results were described in terms of frequencies, percentages, mean, and standard deviation. Chi-square tests and bivariate binary logistic regression analyses were performed to assess the association between individual characteristics and overweight. Multiple regression models were developed using different variable inclusion criteria based on p-value thresholds (<0.1, < 0.2, and <0.3) and compared against a full model guided by theoretical relevance. The final multivariate logistic regression model included variables with p-values less than 0.2 to adjust for potential confounding factors. Model fit was evaluated using the Hosmer-Lemeshow goodness-of-fit test and the Nagelkerke R-squared statistic. Associations were considered statistically significant at a p-value of less than 0.05. Adjusted odds ratios (AORs) with 95% confidence intervals (CIs) were reported to reflect the strength and precision of the observed associations.

### Ethical approval

The approval for the study was obtained from the Institutional Review Committee (IRC) of the Institute of Medicine, Tribhuvan University (Reference number: 348(6–11)E2080/081). Before the day of data collection, participants were requested to obtain written consent from their parents/caregivers. Both verbal and written informed consent were obtained from the participants. Additionally, a written assent form was obtained from the participants who were below 18 years. The right to withdraw from participation was ensured throughout the study. Confidentiality and privacy of participants’ information were maintained and assured.

### Variables and their operational definitions

**Adolescents:** Individuals in the age group of 10–19 years of age were considered adolescents (3). Adolescents of age group 10–13 years, 14–16 years, and 17–19 years were considered as early, middle, and late adolescents, respectively [32].

**Birth order:** The chronological order of a sibling’s birth in a family was considered birth order.

**Physical environment:** Physical environment refers to factors such as access to a playground at school, availability of playgrounds around the home, physical activities at school, and means of going to school.

**Physical activity at school:** It refers to any activities that require energy expenditure and are conducted as part of the school environment, such as physical education classes, sports, yoga, and other extracurricular physical activities.

**Physical activity:** Vigorous, moderate activities were considered as physical activities. According to the WHO, adolescents should do moderate to vigorous physical activity for at least 60 minutes per day [33].

**Vigorous physical activity:** Activity that causes large increases in breathing or heart rate, like carrying or lifting heavy loads, digging, playing sports, cycling a rickshaw, or construction work for at least 10 minutes continuously, was considered vigorous physical activity [30].

**Moderate physical activity:** Those activities that cause small increases in breathing or heart rate, such as brisk walking, carrying light loads, manually washing clothes, mopping the floor, and gardening at home for at least 10 minutes continuously, were considered moderate physical activity [30].

**Eating habits:** The eating habits included the frequency of consuming fruits, vegetables, meat, fish, eggs, fast food, and soft/sweetened drinks per week.

**Fast foods:** Those foods that are easily available and can be bought quickly from restaurants/hotels are considered fast food [34].

**Junk foods:** Those foods that have very low nutritional value and are high in calories, with added sugar, salt, or saturated fat, are considered junk foods [34].

**Sedentary behavior:** The total number of hours adolescents spend sitting per day was considered to assess sedentary behavior. The sitting hours above 6 hours were considered as adolescents having sedentary behavior [35].

**Screen time:** Watching television and playing video games or using electronic devices for more than 2 hours per day was defined as excessive screen time [36].

**Overweight** Adolescents with z-score>+1 SD (equivalent to BMI 25 kg/m^2^ at 19 years) were considered as overweight [37].

## Results

### Social and demographic characteristics of participants

There was a total of 282 participants in the study. The mean ± SD age was 16.8 ± 0.1 years. Most participants were Hindu (77%) and half were Janajati (50.4%) by ethnicity. One-third of the participants were from government schools. Around 61% of participants belonged to the nuclear family (Table 1).

**Table 1:** Social and demographic characteristics of participants.

Characteristics	Number (n)	Percentage (%)
**Age (in completed years)**		
Mean ± SD	16.8 ± 0.98	
14-16	111	39.4
17-19	171	60.6
**Sex**		
Female	156	55.3
Male	126	44.7
**Religion**		
Hindu	217	77.0
Buddhist	57	20.2
Others (Christian, Kirat)	8	2.8
**Ethnicity**		
Janajati	142	50.4
Brahmin/Chhetri	116	41.1
Dalit	24	8.5
**Birth order**		
First	137	48.6
Middle	53	18.8
Last	92	32.6
**School type**		
Private	187	66.3
Government	95	33.7
**Family type**		
Nuclear	171	60.6
Joint/extended	111	39.4
**Mother’s education**		
Illiterate	115	40.9
Literate	167	59.1
**Father’s education**		
Illiterate	72	25.8
Literate	210	74.2
**Occupation of the mother**		
Housemaker	147	52.0
Agriculture	55	19.9
Private job/business	56	19.6
Others (foreign employment, labor, government service)	24	8.5
**Occupation of the father**		
Private job/business	121	43.4
Agriculture	49	16.5
Government service	43	15.4
Others (foreign employment, labor)	69	24.7
**House type**		
Rent	176	62.4
Own house	106	37.6

### Physical environment and behavior-related characteristics

Around 90% and 85% had access to a playground and physical activities at school, respectively. Two-thirds (66%) used to go to school on walking. Nearly half of the participants were involved in vigorous activity (45%) and moderate activity (47.5%), respectively. The majority (95.7%) spent 6 or fewer hours per day in sedentary behavior, while 55.3% spent more than two hours daily on-screen time (Table 2).

**Table 2:** Physical environment and behavior-related characteristics of participants.

Characteristics	Number (n)	Percentage (%)
**Playground at school**		
Yes	254	90.1
No	28	9.9
**Physical activities at school**		
Yes	240	85.1
No	42	14.9
**Playground around home**		
Yes	174	61.7
No	108	38.3
**Means of going to school**		
Walking	186	66.0
Vehicles	96	34.0
**Vigorous activity**		
Yes	127	45.0
No	155	55.0
**Time of doing vigorous activity per day, (n = 127)**		
<60 min	75	59.1
>60 min	52	40.9
**Moderate activity**		
Yes	134	47.5
No	148	52.5
**Time of doing moderate activity per day, (n = 134)**		
<60 min	79	59.0
≥60 min	55	41.0
**Duration of sedentary behavior (per day)**		
≤ 6 hours	270	95.7
> 6 hours	12	4.3
**Duration of screen time (per day)**		
≤2 hours	126	44.7
. > 2 hours	156	55.3
**BMI**		
Normal	244	86.6
Overweight	38	13.4

### BMI characteristics of participants

**Fig 2:**
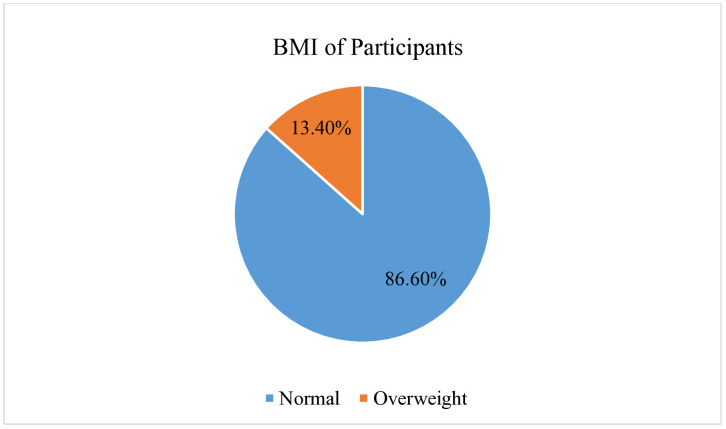
BMI Characteristics of participants.

This study found that 86.6% (n = 244) and 13.4% (n = 38) were normal and overweight, respectively (Fig 2).

### Food habits of participants

Most participants (98.2%) had a diet primarily consisting of rice and pulses. Most of the participants consumed (52.5%) three meals per day. Half of the participants (50.4%) skipped meals 1–3 times per week. Among them, breakfast was the most commonly skipped meal (36.2%). About 57% consumed fast food 1–3 times per week, and 32.3% consumed it more than three times per week. The main source of lunch during school time was the school canteen (69.9%). Most of the participants (78%) received pocket money for lunch at school (Table 3).

**Table 3:** Food habits of participants.

Characteristics	Number (n)	Percentage (%)
**Main diet**		
Rice -Pulses	277	98.2
Wheat/Maize	5	1.8
**Frequency of meals per day**		
Less than 3 times	93	33.0
Equals to 3 times	148	52.5
More than 3 times	41	14.5
**Frequency of skipping meals per week**		
Never	119	42.2
1-3 times	142	50.4
More than 3 times	21	7.4
**Type of skipped meals per week**		
Breakfast	59	36.1
Lunch	25	15.3
Day Snacks	37	22.8
Dinner	42	25.8
**Consumption of green leafy vegetables per week**		
Never	11	3.9
1-3 times	166	58.9
More than 3 times	105	37.2
**Consumption of fruits per week**		
Never	28	9.9
1-3 times	187	66.3
More than 3 times	67	23.8
**Consumption of meat per week**		
Never	28	9.9
1-3 times	190	67.4
More than 3 times	64	22.7
**Consumption of fish per week**		
Never	125	44.3
1-3 times	134	47.5
More than 3 times	23	8.2
**Consumption of eggs per week**		
Never	35	12.4
1-3 times	178	63.1
More than 3 times	69	24.5
**Consumption of fast food per week**		
Never	30	10.6
1-3 times	161	57.1
More than 3 times	91	32.3
**Consumption of junk food per week**		
Never	34	12.1
1-3 times	149	52.8
More than 3 times	99	35.1
**Consumption of sweetened drinks per week**		
Never	36	12.8
1-3 times	163	57.8
More than 3 times	83	29.4
**Main source of food during lunch hour at school**		
School canteen	197	69.9
Homemade	65	23.0
Others (street food, restaurant, supermarket)	20	7.1
**Pocket money for lunch at school**		
Yes	220	78.0
No	62	22.0
**Amount of pocket money for lunch (NRs.)**		
≤ NRs.100	192	87.3
> NRs.100	28	12.7

### Association between individual characteristics and overweight

The table depicts that factors such as school type (AOR: 2.6, CI: 1.9–8.2), father’s education (AOR: 2.1, CI: 1.7–6.5), access to physical activity at school (AOR: 1.2, CI: 1.1–4.6), and pocket money for lunch at school (AOR: 0.3, CI: 0.2–0.5) were found to be significantly associated with overweight (Table 4).

**Table 4:** Factors associated with overweight among the participants.

	Bivariate analysis				Multivariate logistic regression	
Characteristics	Overweight		p-value	COR (95%CI)	p-value	AOR (95%CI)
	Yes n (%)	No n (%)				
**Age**						
14-16	17 (15.3)	94 (84.7)	0.467	Ref		
17-19	21 (12.3)	150 (87.7)		0.7 (0.4-1.5)		
**Sex**						
Male	16 (12.7)	110 (87.3)	0.731	Ref		
Female	22 (14.1)	134 (85.9)		1.1 (0.6- 2.3)		
**Religion**						
Hindu	27 (12.4)	190 (87.6)	0.355	Ref		
Others	11 (16.9)	54 (83.1)		1.4 (0.7- 3.1)		
**Ethnicity**						
Brahmin/Chhetri	16 (13.8)	100 (86.2)	0.418	Ref		
Janajati	21 (14.8)	121 (85.2)		1.1 (0.5- 2.2)		
Dalit	1 (4.2)	23 (95.8)		0.3 (0.0- 2.2)		
**Family type**						
Nuclear	26 (15.2)	145 (84.8)	0.293	Ref		
Joint/extended	12 (10.8)	99 (89.2)		0.6 (0.3-1.4)		
**School type**						
Government	5 (5.3)	90 (94.7)	0.004	Ref	0.047	Ref
Private	33 (17.6)	154 (82.4)		3.8 (1.5- 10.2)		2.6 (1.9- 8.2)
**Birth order**						
First	15 (10.9)	122 (89.1)	0.476	Ref		
Middle	8 (15.1)	45 (84.9)		1.4 (0.6- 3.6)		
Last	15 (16.3)	77 (83.7)		1.5 (0.7- 3.4)		
**Mother’s education**						
Illiterate	14 (12.2)	102 (87.8)	0.357	Ref		
Literate	24 (14.5)	142 (85.5)		0.8 (0.4- 1.2)		
**Father’s education**						
Literate	6 (6.9)	69 (93.1)	0.046	Ref	0.018	Ref
Illiterate	32 (15.5)	175 (84.5)		1.4 (1.2- 2.1)		2.1 (1.7- 6.5)
**Occupation of the mother**						
House maker	21(14.4)	125 (85.6)	0.724	Ref		
Agriculture	5 (9.1)	50 (90.9)		0.6 (0.2- 1.7)		
Private job/ business	9 (16.1)	48 (83.9)		1.1 (0.5- 2.7)		
Others	3 (12.5)	21 (87.5)		0.8 (0.2- 3.1)		
**Occupation of the father**						
Agriculture	3 (6.5)	43 (93.5)	0.184	Ref	0.180	Ref
Government service	4 (7.0)	41 (93.0)		1.1 (0.2- 5.6)		0.2 (0.0- 1.6)
Private job/business	20 (16.5)	102 (83.5)		2.8 (0.8- 10.1)		0.8 (0.2- 3.5)
Others	11 (15.9)	58 (84.1)		2.7 (0.7- 10.3)		1.2 (0.3- 5.6)
**House type**						
Own house	18 (17.0)	88 (83.0)	0.183	Ref	0.308	Ref
Rent	20 (11.4)	156 (88.6)		0.6 (0.3-1.2)		0.6 (0.3-1.5)
**Physical activity at school**						
Yes	25 (9.9)	215 (91.1)	0.019	Ref	0.021	Ref
No	13 (30.9)	29 (69.1)		3.8 (1.2- 7.6)		1.2 (1.1- 4.6)
**Means of going to school**						
Walking	21 (11.3)	165 (88.7)	0.144	Ref	0.667	Ref
Vehicles	17 (17.7)	79 (82.3)		1.7 (0.8- 3.4)		1.2 (0.5- 2.6)
**Vigorous activities**						
Yes	17 (13.4)	110 (86.6)	0.554	Ref		
No	21 (13.5)	134 (86.5)		1. (0.5- 2.0)		
**Moderate activities**						
Yes	17 (12.7)	117 (87.3)	0.424	Ref		
No	21 (14.2)	127 (85.8)		1.1 (0.6- 2.3)		
**Duration of sedentary behavior (per day)**						
≤ 6 hours	35 (13.0)	235 (87.0)	0.379	Ref		
> 6 hours	3 (25.0)	9 (75.0)		2.2 (0.6- 8.7)		
**Duration of screen time (per day)**						
≤2 hours	13 (10.3)	113 (89.7)	0.219	Ref		
. > 2 hours	25 (16.0)	131 (84.0)		1.6 (0.9- 3.4)		
**Frequency of meals per day**						
Less than 3 times	13 (14.0)	80 (86.0)	0.189	Ref	0.305	Ref
Equal to 3 times	16 (10.8)	132 (89.2)		0.7 (0.3- 1.6)		0.7 (0.3- 1.8)
More than 3 times	9 (22.0)	32 (78.0)		1.7 (0.7- 4.4)		1.7 (0.6- 5.2)
**Frequency of skipping meals per week**						
Never	11 (9.2)	108 (90.8)	0.202	Ref		
1-3 times	23 (16.2)	119 (83.8)		1.8 (0.9- 4.1)		
More than 3 times	4 (19.0)	17 (81.0)		2.3 (0.7- 8.1)		
**Frequency of junk consumption per week**						
Never	6 (17.6)	28 (82.4)	0.528	Ref		
1-3 times	17 (11.4)	132 (88.6)		0.6 (0.2- 1.7)		
More than 3 times	15 (15.2)	84 (84.8)		0.8 (0.3 - 2.4)		
**Pocket money for lunch at school**						
Yes	35 (15.9)	185 (84.1)	0.033	Ref	0.041	Ref
No	3 (4.8)	59 (95.2)		0.2 (0.1- 0.6)		0.3 (0.2- 0.5)

*Ref = reference category*

* *p-value<0.05*

## Discussion

Overweight among adolescents has become a problem in LMICs like Nepal in recent years [9,14]. This study found that 13.4% of adolescents were overweight, which is similar to a study conducted in Bangladesh 13.5% [18]. A similar finding is also reported in different areas of Nepal, like Lalitpur (12.2%) and Rasuwa (14.6%) districts [38,39]. In contrast, several studies conducted among adolescents in the UAE (18.3%) [16], Morocco (29%) [40], Mexico (26.5%) [41], and India (19%) [17] Ajman, Sharjah and Dubai 18.3% [16], Delhi 19% [17] reported a higher prevalence of overweight compared to our study. The notable prevalence of overweight adolescents in Nepal is due to the high consumption of junk food, cultural differences, and their food choices, and the adoption of a sedentary lifestyle among adolescents [16,17,41]. Furthermore, culture significantly shapes dietary behaviors, physical activity, and perceptions of body image among adolescents, thereby influencing overweight and obesity risk [17,41]. Traditional food practices may promote high-calorie intake, while cultural norms around body size can normalize or even idealize overweight [17]. Similarly, a higher prevalence of overweight was observed in different regions of Nepal, i.e., Tokha (45.2%) [42], and Tulsipur (18%) [43]. This concludes that adolescents in urban areas are more likely to be overweight due to greater access to junk and processed foods, increased screen time, and reduced physical activity [31,44]. Urban lifestyles often involve sedentary behavior, reliance on motorized transport, and limited open spaces for exercise. Higher socio-economic status also contributes to unhealthy dietary choices, while academic pressure and easy availability of screen-based entertainment further reduce physical activity [44].

Likewise, a lower prevalence of overweight was reported in studies conducted in Bosnia (6.5%) [45], and Pakistan (8%) [46]. This might be due to community initiatives and awareness campaigns launched at schools in these countries about the risk of overweight [44]. However, our study finding is notably higher compared to the study conducted in Lalitpur (9.34%) [35], Bharatpur (9.8%) [47], and National Adolescent Nutrition Survey, 2014 (1%) [6].

This study demonstrated a higher prevalence of overweight among late adolescents as compared to middle adolescents. This finding is supported by the previously conducted study in Nagarjun [48] and Rasuwa [39]. This could be due to significant hormonal changes and the completion of growth spurts during late adolescence, leading to a decrease in metabolic rate and increased fat accumulation [47]. Also, the transition to a more sedentary lifestyle marked by increased screen time and decreased physical activity is more observed in older adolescents as compared to early adolescents [49]. Meanwhile, this finding contrasts with the study conducted in Lalitpur [35] where early adolescents were found to be overweight compared to late adolescents.

This study found higher odds of being overweight among female adolescents than male adolescents and is consistent with the studies conducted in Morocco [40], Egypt [50], Rasuwa [39], and Lalitpur [35]. This might be due to early puberty among females, which is associated with shorter height and higher BMI, which leads to the risk of overweight [39,51]. Another reason might be due to less involvement of female adolescents in physical activity as compared to male adolescents, which results in higher overweight [52]. This study contrasts with another study conducted in Lalitpur [16], where males were more likely to be overweight than female adolescents.

Adolescents having sedentary behaviour with more than 6 hours were found to be more likely to be overweight as compared to their counterparts. This finding is supported by the studies conducted in the UAE [16], Lalitpur [35], Rasuwa [39] and Kathmandu [25]. This could be attributed to lower levels of physical activity, increasing the risk of weight accumulation due to reduced energy expenditure [25].

This study showed that the prevalence of overweight was higher among adolescents who had a screen time of more than 2 hours. Similar findings were reported from the studies conducted in Sri Lanka [53], China [54], India [55] and Nepal [16]. Prolonged screen time reduces physical activity and often leads to increased snacking on high-calorie foods, which exacerbates caloric intake, contributing to weight gain. Additionally, the influence of advertisements for junk and fast food on social media/TV is also a factor that contributes to weight gain [56]. Adolescents who ate more than three meals a day were found to have a higher likelihood of being overweight compared to those who consumed three or fewer meals daily. In some cases, additional “meals” may include unhealthy snacks or junk food high in sugars and fats. These foods are typically low in nutritional value and high in calories, contributing to weight gain [53]. This finding is consistent with a similar study conducted in Kathmandu [25], Kaski [57] and Nagarjun [48].

Adolescents whose fathers were illiterate had 2.1 times higher odds of being overweight compared to those whose fathers were literate. This finding is supported by the studies conducted in Denmark [58], Humla [59] and Jajarkot [60]. This is because educated fathers are more aware of the risks associated with being overweight and guide their children in maintaining appropriate BMI and healthy lifestyles [58,61]. Adolescents who did not engage in physical activity at school were more likely to be overweight than their counterparts. This finding aligns with the studies conducted in the UAE [16], Nagarjun [48] and Rupandehi [22]. Regular physical activity helps burn calories, which contributes to maintaining a balance between calorie intake and expenditure, and without sufficient activity, excess calories are stored as fat, leading to weight gain [22].

Adolescents studying at private schools had 2.6 times higher odds of being overweight compared to those in government schools. This finding is in line with the study conducted in Lalitpur [38] Tulsipur [43], and Dharan [62]. This is due to the higher socio-economic status and greater purchasing power for junk foods among adolescents in private schools [43].

Adolescents who were given pocket money for lunch at school were more likely to be overweight than those who brought homemade lunch to school. This is because students with pocket money tend to purchase unhealthy snacks or fast food, which are high in calories, sugar, and unhealthy fats, contributing to weight gain [26]. This finding is coherent with the study conducted in China [63], Kaski [64] and Nepal [65].

This study highlights several challenges in addressing adolescent overweight in Nepal. There is limited integration of school-based health programs, including regular physical activity and nutrition education. Weak enforcement of policies regulating junk food sales around schools further exacerbates the issue. Socio-economic disparities and sedentary lifestyles driven by increased screen time and reduced physical activity are prominent, especially in urban settings. Moreover, inadequate recreational infrastructure in schools and low health literacy among parents and adolescents hinder effective prevention and control efforts.

## Strengths and limitations of the study

As this study was conducted in one of Nepal’s largest metropolitan areas, its results may be more generalizable to adolescents across the country. The findings could provide valuable evidence to support school-based interventions or programs aimed at combating non-communicable diseases (NCDs).

Obesity and underweight are prevalent nutritional issues in Nepal; however, this study focuses specifically on overweight among adolescents. The self-administered questionnaire may have introduced reporting bias. There might be a chance of recall or social desirability bias among the factors, such as dietary habits and physical activity. Although anthropometric measurements were taken using standardized procedures, they were limited to a single time point, which may not reflect day-to-day variations. Additionally, some potential confounding factors, such as family history of obesity and comprehensive socio-economic indicators, were not assessed, which may affect the interpretation of associations. Finally, the cross-sectional nature of the study restricts the ability to infer causal relationships between the identified factors and overweight.

## Conclusion

This study revealed that 13.4% of adolescents in Kathmandu Metropolitan City are overweight. Key factors significantly associated with being overweight included the type of school attended, the father’s level of education, physical activity at school, and pocket money allocated for lunch. Additionally, late adolescents (ages 16–19) were found to have a higher likelihood of being overweight compared to middle adolescents. These findings underscore the importance of implementing targeted, school-based health interventions aimed at promoting healthy eating habits and increasing physical activity, particularly among older adolescents. Cost-effective interventions should be promoted to address specific risk factors, including educating parents on proper nutrition, regulating adolescents’ pocket money to limit unhealthy food purchases, and incorporating structured physical activities into school programs.

## Supporting information

S1 Dataset Data for analysis in this study.
(XLSX)
